# Disruption of metazoan gene regulatory networks in cancer alters the balance of co-expression between genes of unicellular and multicellular origins

**DOI:** 10.1186/s13059-024-03247-1

**Published:** 2024-04-29

**Authors:** Anna S. Trigos, Felicia Bongiovanni, Yangyi Zhang, Maia Zethoven, Richard Tothill, Richard Pearson, Anthony T. Papenfuss, David L. Goode

**Affiliations:** 1 Peter MacCallum Cancer Centre, 305 Grattan St., Melbourne, VIC 3000 Australia; 2 Sir Peter MacCallum Department of Oncology, The University of Melbourne, Parkville, VIC 3010 Australia; 3 Department of Biochemistry and Molecular Biology, Monash University, Clayton, VIC 3168 Australia; 4 Centre for Cancer Research, The University of Melbourne, Parkville, VIC 3010 Australia; 5 Department of Biochemistry and Molecular Biology, The University of Melbourne, Parkville, VIC 3010 Australia; 6 Bioinformatics Division, The Walter & Eliza Hall Institute of Medical Research, Parkville, VIC 3052 Australia

**Keywords:** Cancer, Gene expression, Expression modules, Atavism, Evolution

## Abstract

**Background:**

Metazoans inherited genes from unicellular ancestors that perform essential biological processes such as cell division, metabolism, and protein translation. Multicellularity requires careful control and coordination of these unicellular genes to maintain tissue integrity and homeostasis. Gene regulatory networks (GRNs) that arose during metazoan evolution are frequently altered in cancer, resulting in over-expression of unicellular genes. We propose that an imbalance in co-expression of unicellular (UC) and multicellular (MC) genes is a driving force in cancer.

**Results:**

We combine gene co-expression analysis to infer changes to GRNs in cancer with protein sequence conservation data to distinguish genes with UC and MC origins. Co-expression networks created using RNA sequencing data from 31 tumor types and normal tissue samples are divided into modules enriched for UC genes, MC genes, or mixed UC-MC modules. The greatest differences between tumor and normal tissue co-expression networks occur within mixed UC-MC modules. MC and UC genes not commonly co-expressed in normal tissues form distinct co-expression modules seen only in tumors. The degree of rewiring of genes within mixed UC-MC modules increases with tumor grade and stage. Mixed UC-MC modules are enriched for somatic mutations in cancer genes, particularly amplifications, suggesting an important driver of the rewiring observed in tumors is copy number changes.

**Conclusions:**

Our study shows the greatest changes to gene co-expression patterns during tumor progression occur between genes of MC and UC origins, implicating the breakdown and rewiring of metazoan gene regulatory networks in cancer development and progression.

**Supplementary Information:**

The online version contains supplementary material available at 10.1186/s13059-024-03247-1.

## Background

Changes to transcriptional regulation played a major role in the evolution of complex multicellularity [1, 2]. Early metazoan species inherited genes from their unicellular ancestors that underpinned core biological functions such as cell division, motility, and metabolism. Stable multicellularity required fine control over where and when such processes occurred. Expanded gene regulatory networks (GRNs) evolved to coordinate the activity of unicellular genes and sustain the cooperative growth crucial for functional multicellularity [3, 4]. The essentiality of unicellular genes for cellular homeostasis meant a simple on/off mode of regulation would not suffice. Metazoans need to maintain the expression of unicellular genes at safe levels and periodically upregulate them for development, wound healing, stress responses, and other processes. Metazoan GRNs evolved to balance the expression of genes with unicellular and multicellular origins, leading to their consistent co-expression across healthy tissues [5, 6].

Many hallmarks of cancer involve unraveling of core features of multicellularity [7], including reduced cell–cell adhesion, lack of coordinated cell division, altered metabolism, and breakdown of tissue differentiation. The atavism hypothesis of cancer proposes reactivation of conserved transcriptional programs inherited from ancestral unicellular species enhances the adaptability and replicative potential of tumor cells, linking changes in gene regulation to loss of multicellularity in cancer [8–11].

Increasing molecular evidence supports a role for changes to the balance of expression between unicellular and multicellular genes during the formation and progression of cancer. In earlier work, we found pervasive downregulation of genes of multicellular origin and selective upregulation of genes of unicellular origin across solid cancers [5, 12], since independently confirmed [13]. Similar approaches showed both genetic and transcriptional alterations accumulate in genes involved in multicellularity during selection for accelerated growth and metastatic potential in breast cancer xenografts [14], in highly conserved genes in myeloma upon exposure to chemotherapy [15] and in recently evolved paralogs of cell cycle control genes in cancer cell lines [16]. An example of the functional impacts is the stress-induced activation of error-prone DNA repair mechanisms conserved with bacteria in a range of tumors, facilitating drug resistance [17, 18], and leaving detectable mutational signatures [19, 20].

Human protein–protein interaction (PPI) and gene regulatory networks show layering of genes by evolutionary age, with highly conserved genes of unicellular origin found closer to the center and more recently evolved genes on the peripheries [21, 22]. This creates structural vulnerabilities that can lead to cancer [23]. We found transcriptional regulators forming hubs connecting genes with unicellular origins with genes of multicellular origins in human GRNs are frequently enriched for somatic mutations in cancer [24], implicating the dysfunction of such hubs as important drivers of tumor formation and progression. Intriguingly, somatic amino acid substitutions in known cancer driver genes sometimes match the dominant or fixed allele in unicellular eukaryotes [25–27] causing direct reversion to ancestral protein sequences that may alter oncogenic pathways.

Here, we investigate how the disruption of metazoan GRNs leads to an imbalance in the expression of unicellular and multicellular genes in cancer. First, protein sequence orthology data were used to assign evolutionary age categories to human genes. Genes conserved in multiple single-celled species were placed in the unicellular category. Otherwise, genes were labeled as being multicellular if orthologs could only be found in other metazoan species. We performed gene co-expression analysis to infer gene regulatory networks for 31 types of solid cancers. Overlaying evolutionary gene age categories onto co-expression networks found major changes in the co-expression of unicellular and multicellular genes occurs during tumor formation and progression. Unicellular and multicellular genes not usually co-expressed in normal tissues come together to form distinct co-expression modules in tumors that often include known cancer driver genes. The activity and degree of rewiring within these modules increase with both tumor grade and degree of malignancy.

Altogether, disruption and rewiring of metazoan GRNs are common and important aspects of tumor progression that causes an imbalance in the co-expression of unicellular and multicellular genes. Our framework demonstrates a new way to combine sequence conservation and co-expression analysis to study the links between the evolution, multicellularity, and cancer.

## Results

### Tracking changes in co-expression between unicellular and multicellular genes in cancer

**Fig. 1:**
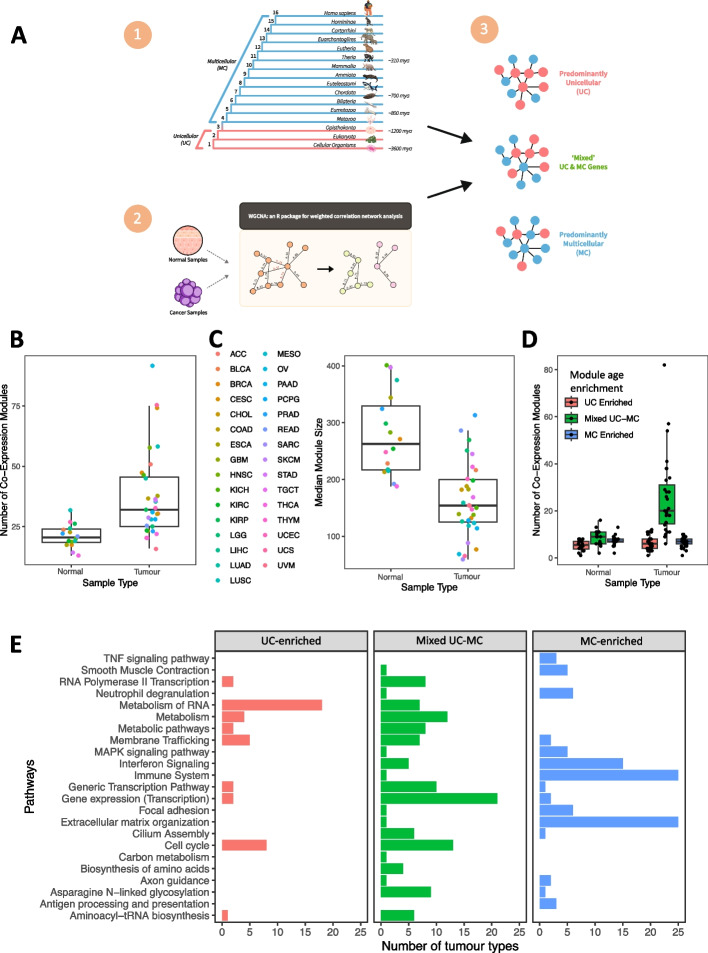
WGCNA-derived gene co-expression modules in tumor and normal samples. **A** Identification and classification of gene co-expression modules. (1) Phylostratigraphy was applied to assign unicellular or multicellular origins to genes. (2) WGCNA was used to cluster genes into co-expression modules based on correlation in expression. (3) Modules were classified according to the degree of enrichment of genes of unicellular or multicellular origin. **B** Summary of number of modules per TCGA cohort, with points colored according to tumor type. **C** Mean number of genes in tumor and normal modules per TCGA cohort. Color key is the same as in (**B**). **D** Distribution of numbers of unicellular-enriched, multicellular-enriched, and mixed UC-MC modules across TCGA cohorts. **E** KEGG and reactome terms found to be most frequently enriched in modules across all TCGA cohorts. The total numbers of tumor types containing a unicellular, mixed UC-MC, or multicellular module enriched for each term are shown on the *x*-axis

Alterations to the structure of metazoan GRNs that evolved to support multicellularity should manifest as differences in patterns of co-expression of unicellular and multicellular genes in tumors relative to normal somatic tissues. To investigate, we constructed a three-step pipeline to combine sequence orthology data with gene co-expression networks to study how genes of unicellular or multicellular origin are co-regulated in cancer (Fig. 1A).

We first assigned evolutionary ages to human genes using phylostratigraphy, which employs phylogenetics and multiple sequence alignments to estimate the point in evolutionary time where a gene emerged [6]. Phylostratigraphic analysis revealed cancer genes tend to be highly conserved and enriched in particular for genes that emerged in unicellular organisms and during the early stages of metazoan evolution [28]. We divided species into 16 clades representing the major stages of evolution from single-celled organisms to modern humans (Fig. 1A (1)), as we have previously done [5]. Briefly, for each human gene, protein sequence alignments from OrthoMCL [29] were used to identify the most distant clade from humans in which species with high-confidence orthologs could be found (the “Methods” section). Genes with orthologs in bacteria or single-cell eukaryotes (clades 1–3) were designated as having unicellular origins (UC genes). Genes with orthologs in other multicellular species only were classified as being of multicellular origin (MC genes). Clade assignments are provided in Additional file 1: Dataset S1.

Next, we inferred the structure and topology of GRNs in both tumors and normal somatic tissues by applying the weighted gene co-expression network analysis (WGCNA) algorithm [30]. WGCNA identifies “modules” of genes with correlated expression across multiple samples (Fig. 1A (2)), implicating underlying coordinated regulatory mechanisms exist that connect those genes into identifiable co-expression modules. WGCNA co-expression modules were generated for each of the 31 solid tumor types with available RNA sequencing data in The Cancer Genome Atlas (TCGA). For the 16 TCGA cohorts with data for at least 10 normal samples, we generated both tumor and normal gene co-expression modules (bladder urothelial carcinoma (BLCA), breast invasive carcinoma (BRCA), colon adenocarcinoma (COAD), esophageal carcinoma (ESCA), head and neck squamous cell carcinoma (HNSC), kidney chromophobe (KICH), kidney renal clear cell carcinoma (KIRC), kidney renal papillary cell carcinoma (KIRP), liver hepatocellular carcinoma (LIHC), lung adenocarcinoma (LUAD), lung squamous cell carcinoma (LUSC), prostate adenocarcinoma (PRAD), rectum adenocarcinoma (READ), stomach adenocarcinoma (STAD), thyroid carcinoma (THCA), uterine corpus endometrial carcinoma (UCEC)). Here, gene expression data from matched pairs of tumor-normal samples were used to identify co-expression modules. For cohorts with no normal samples available, only tumor co-expression modules were built, using gene expression data from all available tumor samples.

Finally, co-expression modules were classified according to the prevalence of unicellular or multicellular genes (Fig. 1A; the “Methods” section) into three categories. Modules enriched in UC genes were defined as being unicellular-enriched, while modules enriched in MC genes were defined as multicellular-enriched. Modules not enriched in either UC or MC genes were placed into the mixed UC-MC category and represent regions of human GRNs where there is communication between genes of different evolutionary ages.

In total, 16 to 92 modules were obtained per TCGA tumor-type cohort, for a total of 1503 modules (337 from normal tissues and 1166 from tumor tissues). On average, there were more modules in tumor samples (37.61 per cohort) than in normal samples (21.06 per cohort) (Fig. 1B) (two-sided Wilcoxon test *p*-value = 4.77 × 10^−5^). This trend held even when only cohorts with matched tumor and normal samples were considered (two-sided Wilcoxon test *p* = 6.87 × 10^−5^) (Additional file 2: Fig. S1). The number of modules detected per cohort was not a function of sample size (Additional file 2: Fig. S2). Tumor modules were typically about half the size of normal modules, with an average of 163.4 genes per tumor module versus an average of 278.1 genes per normal module (two-sided Wilcoxon test *p*-value = 1.63 × 10^−5^) (Fig. 1C). Again, this was true when comparing only cohorts with matched normal samples (two-sided Wilcoxon test *p*-value = 6.87 × 10^−5^) (Additional file 2: Fig. S3) and was independent of cohort size (Additional file 2: Fig. S2). Generally, the modules obtained from WGCNA provide comprehensive coverage of the transcriptome from TCGA cohorts, with more than 90% of genes assigned to modules in 28 of 31 tumor types (Additional file 2: Fig. S4). These patterns are consistent with the greater heterogeneity seen between tumors and the breakdown of links between different GRN regions in cancer.

The higher number of co-expression modules derived from tumor samples was due to an excess of modules belonging to the mixed UC-MC category, which contains a balanced mix of unicellular and multicellular genes. An average of 24.97 mixed UC-MC modules were obtained per TCGA tumor cohort, ranging from 6 to 82 per tumor type (Fig. 1D, Additional file 2: Table S1). Collectively, 774 of the 1166 (66.38%) modules found across all tumor cohorts combined were mixed UC-MC. In marked contrast, only 137/337 (40.65%) of the combined modules from all normal cohorts together were mixed UC-MC (Additional file 2: Fig. S5) (Fisher exact test *p* = 4.87 × 10^−17^, odds ratio = 2.88 (2.23–3.73)). Unicellular-enriched modules comprised 185/1166 (15.87%) of modules from tumor samples whereas 207/1166 (17.75%) of tumor modules were multicellular-enriched (Additional file 2: Table S1). In contrast, 84/337 (24.93%) modules from normal cohorts were unicellular-enriched while 116/337 (34.42%) were multicellular-enriched, significantly different proportions from tumor modules (Fisher exact test *p* = 1.46 × 10^−16^). Most tumor cohorts had similar numbers of unicellular-enriched (mean of 5.97) and multicellular-enriched (mean of 6.68) modules, comparable to normal cohorts, which averaged 5.25 unicellular-enriched and 7.25 multicellular-enriched modules, respectively (Fig. 1D and Additional file 2: Table S1). These patterns hold even if the after connections below the median edge weight (i.e., the 50% weakest edges) are removed from each module (Additional file 2: Fig. S6A).

To understand their functional roles, all co-expression modules were tested for enrichment of terms from the Kyoto Encyclopedia of Genes and Genomes [31] and Reactome [32] using gProfiler [33]. Unicellular-enriched modules were associated with basic cellular processes such as translation, metabolism, and cell cycle, consistent with the highly conserved nature of the genes in those modules. Modules from the multicellular-enriched category were largely associated with functions specific to multicellularity, such as immunity and the extracellular matrix (Fig. 1E). Interestingly, mixed UC-MC modules were enriched for terms related to transcription and mRNA processing as well as core processes such as metabolism and cell cycle, consistent with this class of modules representing processes used by metazoans regulate gene expression of unicellular pathways. Shared genes were observed between modules across TCGA cohorts to a greater extent than would be expected by chance (Additional file 2: Fig. S7), further arguing for common biological drivers behind their formation.

Gene co-expression analysis suggests GRNs in cancers can be divided into regions predominantly composed of genes from unicellular or multicellular ancestors or regions connecting both types of genes. The differences in the prevalence and size of mixed UC-MC modules between tumor and normal cohorts suggest the onset of cancer is accompanied by substantial changes in the co-expression of unicellular and multicellular genes.

### Gene co-expression analysis implicates substantial rewiring of regulatory links between unicellular and multicellular genes in cancer

**Fig. 2:**
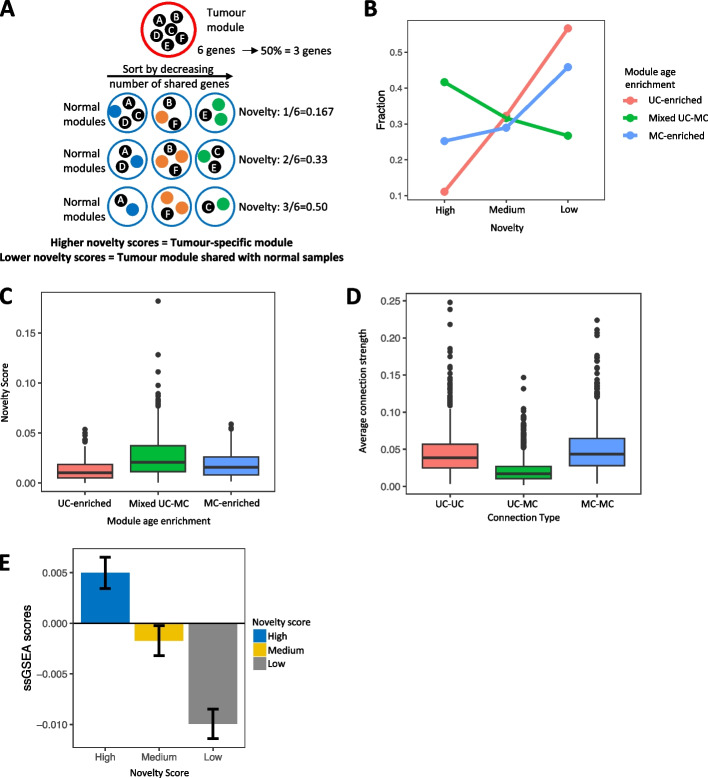
Measuring divergence in co-expression between WGCNA modules from tumors and normal tissues. **A** Demonstration of calculation of module novelty scores based on overlap in gene content between tumor and normal co-expression modules. **B** Fraction of UC-enriched (pink), mixed UC-MC (green), and MC-enriched (blue) modules with high, moderate, or low novelty, combined across all 16 TCGA cohorts with matching normal tissue data. **C** Distribution of novelty scores for tumor modules enriched in unicellular genes (UC-enriched; pink), containing a mix of unicellular and multicellular genes (mixed UC-MC; green) or enriched in multicellular genes (MC-enriched; blue). Data are combined across all 16 TCGA cohorts with matching normal tissue data. **D** Average edge weights for the edges between pairs of unicellular genes (UC-UC; pink), between unicellular and multicellular genes (UC-MC; green), and between pairs of multicellular genes (MC-MC; blue), for all tumor modules from all TCGA cohorts combined. **E** Single-sample GSEA scores for tumor modules with high (blue), medium (yellow), and low (gray) novelty scores, across all 16 TCGA cohorts with matching normal tissue data

To assess the frequency and magnitude of rewiring between UC and MC genes into new co-expression modules, we devised a metric to measure the extent to which modules in tumors contain unique assemblages of genes relative to their matched normal counterparts. Our metric, which we refer to as the “novelty” score, is designed to capture the relative degree of change in the co-expression between conditions represented by a given module. Briefly, it reflects the number of genes within a given tumor module that were also found together in a module from the corresponding normal tissue type (Fig. 2A; the “Methods” section). Based on this score, tumor modules were designated as low, medium, or high novelty based on their overlap with normal modules from the corresponding tissue type. Low novelty modules preserve correlation in expression between tumor and normal tissues; for example, transcriptional programs that retain activity in tumors. Medium or high novelty modules have fewer co-occurring pairs with normal modules and thus better capture co-expression patterns present in tumors but not in the corresponding normal tissue.

Novelty of modules was tightly associated with module age. Overall, 56.66% of all UC-enriched modules and 45.85% of MC-enriched tumor modules across all cohorts were low novelty (Fig. 2B), indicating correlations in expression between pairs of genes that are both from unicellular phylostrata or both from multicellular phylostrata are more likely to be preserved in cancer. Mixed UC-MC tumor modules had higher novelty scores than modules enriched for UC or MC genes (one-sided Wilcoxon test *p*-values = 1.22 × 10^−11^ and 4.80 × 10^−5^, respectively) (Fig. 2C, Additional file 2: Fig. S8). Averaging across cohorts, 41.66% of mixed UC-MC modules were high novelty (Fig. 2B), forming the majority of the high novelty tumor modules identified in tumor cohorts (Additional file 2: Fig. S9). Mixed UC-MC modules still had the highest novelty after connections below the median edge weight were removed from each module (Additional file 2: Fig. S6B).

Mixed UC-MC modules appear to capture new gene co-expression patterns not present in normal tissue, indicating regulatory links between UC and MC genes are disrupted and rewired during tumor development. We hypothesized if there is rewiring of gene regulatory networks during tumor development, there would be substantial heterogeneity in co-expression of UC and MC genes between patients. In agreement, edge weights assigned by WGCNA between UC and MC genes (UC-MC) were weaker than edges between unicellular genes (UC-UC) or between multicellular genes (MC-MC) when we averaged the strength of these connection types in each module (one-sided Wilcoxon *p*-values < 2.2 × 10^−16^ in both cases), indicating higher levels of heterogeneity and/or transcriptional plasticity in the co-expression of unicellular and multicellular genes in cancer (Fig. 2D). Modules in all gene age enrichment categories contain both unicellular and multicellular genes, meaning while connections between unicellular and multicellular genes are enriched in mixed UC-MC modules, they also contribute significantly to UC-enriched and MC-enriched modules (Additional file 2: Fig. S10).

Finally, measuring the combined expression of the genes in each module in tumors using single-sample gene set enrichment analysis (ssGSEA) [34] revealed high novelty modules had the highest ssGSEA scores, followed by medium then low novelty modules (Fig. 2E). On average, in the upper quartile of ssGSEA scores in each tumor type, 31.53% of modules were high novelty modules, 27.03% of medium novelty modules, compared to just 20.79% of low novelty modules (Additional file 2: Fig. S11). This shows high and medium novelty modules result from active gene expression, as opposed to peripheral contamination from noise in the data.

Overall, these findings indicate that the greatest differences in the co-expression between tumors and normal samples occur between genes found in mixed unicellular-multicellular modules. Mixed UC-MC modules display both high levels of expression in tumors and major differences in the co-expression patterns from normal tissues, as evidenced by their higher novelty scores. This fits a model where the balance in co-expression between unicellular and multicellular genes is consistently altered in tumors through changes in the underlying gene regulatory networks.

### The presence of somatic mutations impacts the composition and topology of tumor gene co-expression modules

Cancer progression is defined by the accumulation of somatic mutations that enhance fitness, often by causing dramatic changes to the regulation of transcription. Our prior work showed somatic mutations to key hubs in GRNs disrupt communication between unicellular and multicellular genes [24]. To examine how somatic mutations affect the co-expression of unicellular and multicellular genes, we overlaid data on copy number alterations, coding insertions and deletions (indels), and predicted deleterious point mutations from TCGA onto modules generated by WGCNA.

**Fig. 3:**
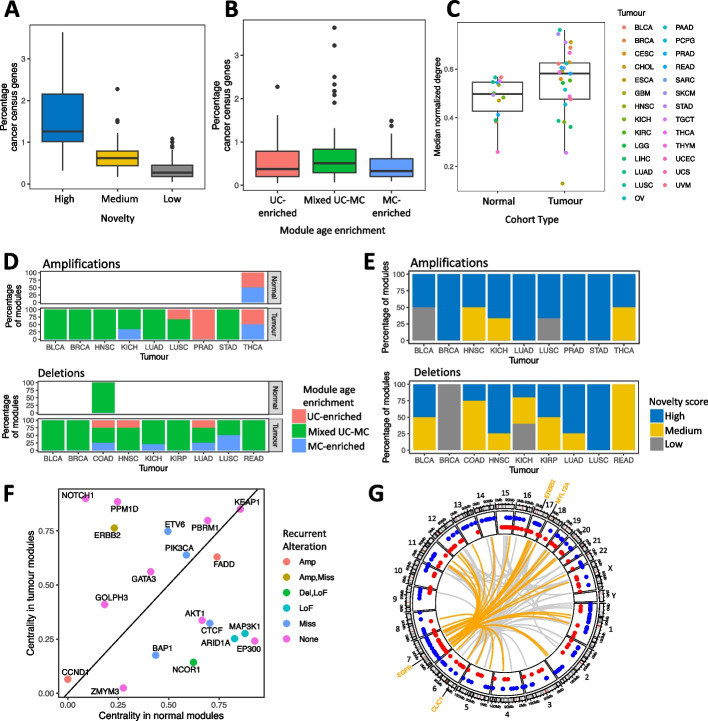
Locations and frequencies of driver genes within tumor gene co-expression modules. **A** Percentages of the COSMIC Cancer Census gene list that are found in high (blue), medium (yellow), or low (gray) novelty modules, across tumor modules with matched normal samples. **B** Percentage of the COSMIC Cancer Census genes found in UC-enriched (pink), mixed UC-MC (green), or MC-enriched (blue) modules, across tumor modules with matched normal samples. **C** Normalized degree of recurrently amplified genes within WGCNA co-expression modules from normal (left) and tumor (right) cohorts, colored according to tumor type. **D** Gene age enrichment distribution for modules where > 10% of genes are recurrently amplified (top) or recurrently deleted (bottom) within the indicated tumor types. Bars show the overall percentages of such modules that are UC-enriched, mixed UC-MC, or MC-enriched. **E** Novelty score distribution for modules where > 10% of genes are recurrently amplified (top) or recurrently deleted (bottom) within the indicated tumor types. **F** Degree centrality in normal modules versus tumor modules for known driver genes in breast cancer. Points colored by the type of recurrent alteration observed in that gene in the BRCA TCGA cohort. **G** CIRCOS plot of the “purple” module from the TCGA low-grade glioma cohort. Points show genomic locations of genes with unicellular (red) or multicellular (blue) origins. Gray lines indicate *cis* and *trans* connections within and between chromosomes for the 100 most strongly co-expressed gene pairs in this module. Locations of the most highly connected genes (EGFR, CLIC1, ERBB2, and MYL12A) are indicated, with the strongest 50 connections to the EGFR oncogene highlighted in orange

First, we calculated the percentage of known cancer drivers from the COSMIC Cancer Census gene list [35] in each tumor co-expression module. We matched genes with the tumor type in which they are considered to be drivers. Novelty score was associated with a fraction of known driver genes, with high novelty modules containing the highest percentage of COSMIC Cancer Census genes (Fig. 3A) compared to medium (one-sided Wilcoxon test *p*-value = 7.01 × 10^−7^) and low novelty modules (*p* = 9.49 × 10^−12^). Furthermore, there was an overall strong correlation between module novelty score and percentage of known cancer drivers (Additional file 2: Fig. S12) (Spearman rho, 0.72; *p*-value = 4.52 × 10^−23^). Gene age enrichment category was also associated with the presence of known cancer genes, as mixed UC-MC modules had a higher percentage of COSMIC Cancer Census genes than UC-enriched or MC-enriched modules (one-sided *p*-values = 0.11 and 0.032, respectively) (Fig. 3B). These findings are consistent with somatic driver mutations leading to the formation of novel gene expression associations between unicellular and multicellular genes.

Given the importance of copy number aberrations (CNAs) in driving gene expression changes in cancer, we assessed the prevalence of recurrently amplified or deleted genes, i.e., those gained/lost in > 10% of patients within a given subtype (the “Methods” section). Tumor modules harboring recurrently amplified genes tended to be more central in the network (i.e., have stronger edge weights) overall than did modules without any recurrently mutated genes (one-sided Wilcoxon test *p*-value = 0.024) (Fig. 3C) suggesting amplifications enhance the strength and consistency across patients of co-expression between unicellular and multicellular genes. In contrast, deletions did not have consistent effects on gene centrality in tumor co-expression modules (Additional file 2: Fig. S13).

The enrichment of recurrently mutated genes in co-expression modules from tumors was primarily due to their associations with amplifications and deletions. Of the 337 modules derived from normal tissues, only 2 (0.59%) were at least 10% of genes recurrently amplified in cancer. In contrast, 51 of 1166 (4.37%) tumor modules contained ≥ 10% genes recurrently amplified in the corresponding cancer type (one-sided Fisher exact *p* = 1.56 × 10^−4^, odds ratio = 0.13 (0.00–0.43)). Likewise, only 1 normal module was enriched in genes affected by deletions in cancer while across all tumor types 39/1166 (3.34%) of co-expression modules contained > 0% recurrently deleted genes (one-sided Fisher exact *p* = 4.32 × 10^−4^, odds ratio = 0.086 (0.00–0.43)). These enrichments are consistent with the capacity for copy number alterations (CNAs) to dramatically elevate or reduce the expression of genes in cancer [36].

The greatest prevalence of recurrent copy number alterations was seen in high novelty modules from the mixed UC-MC category. In seven of nine tumor types with modules with more than 10% of genes harboring recurrent amplifications, the majority of these modules were of the mixed UC-MC category. Similarly, of the modules associated with deletions, the majority were mixed UC-MC in eight of the nine cohorts with modules with more than 10% of genes harboring recurrent deletions (Fig. 3D). Amplifications were also more prevalent in modules with high novelty scores. Across all nine tumor cohorts, high novelty modules comprised 50% or more of the modules containing recurrently amplified genes (Fig. 3E). In contrast, modules with recurrently deleted genes had a broader mix of novelty scores (Fig. 3E). Therefore, some amplifications appear to cause altered co-expression of unicellular and multicellular genes, leading to the formation of high novelty mixed UC-MC modules.

We assessed how the impact of recurrently mutated genes on the transcriptomes in tumors may be mediated by their topological positions within co-expression networks represented by our WGCNA modules. We hypothesized genes driving the formation of distinct co-expression modules in tumors migrate to more central and interconnected positions compared to their original location in normal modules. Conversely, genes involved in the fragmentation of normal modules during tumorigenesis would become less connected and migrate to the periphery of tumor modules. To test this, we calculated the centrality of each gene after normalizing edge weights across modules (the “Methods” section), revealing structural differences between tumor and normal modules.

Marked changes in the centrality of genes between normal and tumor co-expression modules in key driver genes from the breast cancer (BRCA) cohort of TCGA. Multiple known breast cancer driver genes had large changes in centrality (Fig. 3F) that aligned with their established roles in tumorigenesis. Oncogenes (e.g., NOTCH1 and ERBB2) tended to increase in centrality in tumor modules while tumor suppressors (e.g., BAP1, AKT1, and EP300) decreased in centrality. Similar findings emerged in other solid tumor types (Additional file 2: Fig. S14).

Across all cohorts, amplifications had the strongest changes in centrality. Recurrently amplified genes increased centrality in 20 of the 27 TCGA cohorts where they could be found (Additional file 2: Fig. S15) suggesting that upon amplification, they drive form key hubs in distinctive new co-expression modules in tumors. Deletions were also tied to changes in gene centrality, but the impact of gene deletions appears to be context-dependent as the effects on centrality vary by cohort (Additional file 2: Fig. S16). The centrality of recurrently deleted genes was on average reduced from normal tissues to tumors in 15 of the 29 TCGA cohorts examined but increased instead in 10 cohorts. It is important to note here that WGCNA builds modules around both positive and negative correlations in expression between genes [30]. The loss of strong regulators can result in strong negative correlations captured by co-expression patterns specific to certain tumor types.

Gain or loss of large chromosomal segments can lead to simultaneous but coincidental expression changes to multiple linked genes in addition to the selected driver gene or genes [37]. While that may explain some associations between recurrently amplified genes and co-expression modules in tumors, we observed many cases where recurrent amplifications were linked to co-expression changes involving loci across the genome. An example is the “purple” module from the low-grade glioma (LGG) cohort of TCGA, which harbors the EGFR oncogene, a well-known driver of disease progression in glioma [38] (Fig. 3G). Higher activity of this high novelty mixed UC-MC module was associated with poor survival in the LGG cohort (Additional file 2: Fig. S17). The LGG purple module contained genes from every chromosome except chrY and strong co-expression of EGFR with genes across the genome (Fig. 3G). The most highly connected gene in this module is CLIC1, a chloride ion channel with high expression and potential therapeutic and prognostic value in adult glioma [39, 40]. Our work links CLIC1 expression with EGFR activity in LGG.

### The extent of altered co-expression of unicellular and multicellular genes increases during tumor progression

Widespread changes in co-expression patterns between unicellular and multicellular genes in tumors indicate this phenomenon is a general feature of solid cancers. Rewiring of links connecting unicellular and multicellular in GRNs could result in the emergence of many features that accompany tumorigenesis [11, 23]. If so, such changes to the co-expression of unicellular and multicellular genes would be expected to become more pronounced as tumors progress to more malignant states.

To investigate, we tracked the differences in gene co-expression between distinct stages of tumor progression, by sorting samples according to tumor grade and degree of malignancy (Methods). Comparisons were performed in three solid tumor RNA sequencing datasets: (1) data from TCGA prostate cancer (PRAD) cohort and sets of (2) benign skin nevi and melanoma samples [41] as well as (3) malignant pheochromocytomas and benign pheochromocytomas [42]. Gene co-expression modules were generated using WGCNA for each category of samples within each dataset. To assess how gene co-expression changed during tumor progression, we used our novelty score metric to compare modules from high-grade or malignant samples to those from low-grade or benign samples from the same tumor type. Where available, module novelty scores were also computed for benign and low-grade tumors against matched normal tissues, to understand how co-expression changed during the early stages of tumor development. A comparison of gene age enrichments in modules was performed to determine whether the greatest changes in co-expression occurred in UC-enriched, mixed UC-MC, or MC-enriched modules.

**Fig. 4:**
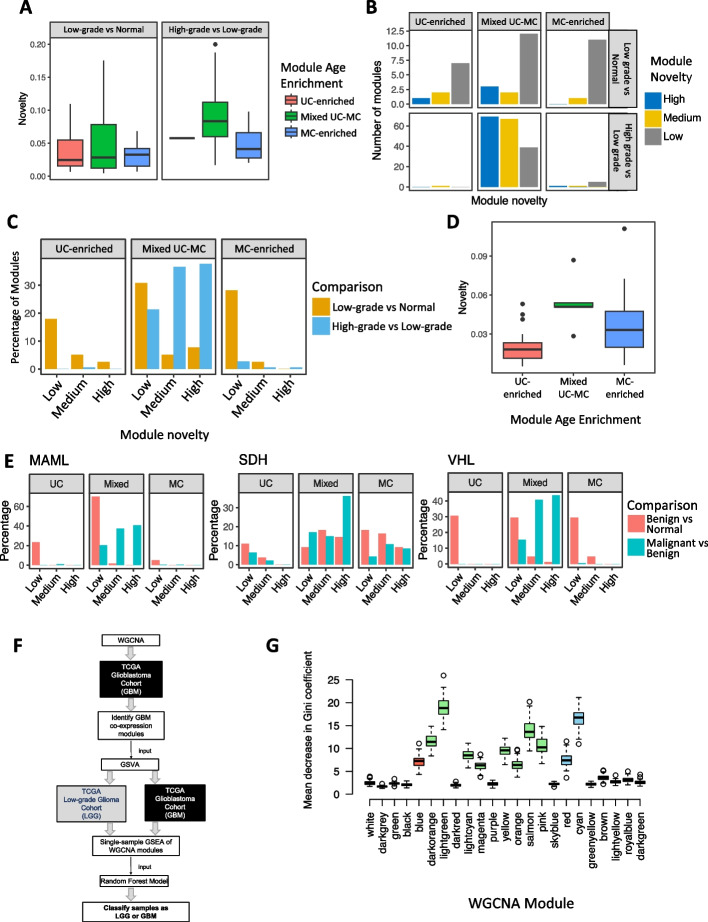
Changes in co-expression between unicellular and multicellular genes during tumor progression. **A** Novelty scores for unicellular-enriched (pink), mixed unicellular-multicellular (green), and multicellular-enriched (blue) co-expression modules derived from low-grade prostate tumors (Gleason grade groups 1 and 2) as compared to co-expression modules from normal (non-diseased) prostate tissue (left) and for co-expression modules from high-grade tumors (Gleason grade groups 4 and 5) as compared to co-expression modules from low-grade prostate tumors (right). Data from TGCA PRAD cohort. **B** Number of UC-enriched, mixed UC-MC, and MC-enriched modules with low, medium, or high novelty scores from low-grade (top) and high-grade (bottom) prostate tumors. **C** Percentage of co-expression modules with low, medium, or high novelty scores from a comparison of low-grade prostate tumors to normal prostate tissues (gold) and from a comparison of high-grade to low-grade prostate tumors (blue), stratified by gene age enrichment category (UC-enriched, mixed UC-MC, or MC-enriched). **D** Novelty score distribution for UC-enriched (pink), mixed UC-MC (green), or MC-enriched (blue) co-expression modules derived from melanoma as compared to co-expression modules derived from benign nevi, using data from Badal et al. **E** Percentage of co-expression modules with low, medium, or high novelty scores for benign tumors compared to normal adrenal gland tissue (pink) and malignant tumors compared to benign tumors (blue), stratified by gene age enrichment category and pheochromocytoma subtype. **F** Schematic of the process of using random forest model to classify adult glioma samples as LGG or GBM based on ssGSEA score of WGCNA modules derived from the TCGA glioblastoma cohort. **G** Power of co-expression modules derived from the TCGA glioblastoma cohort to distinguish low-grade from glioblastoma samples, expressed as distribution of mean decrease in Gini coefficient for 100 replicates of a random forest model. Gene age enrichment category is indicated by color: UC-enriched (pink), mixed UC-MC (green), or MC-enriched (blue)

For the TCGA PRAD cohort, novelty scores were calculated for modules from high-grade primary tumors (annotated as grade groups 4 and 5) compared to the modules derived from low-grade primary tumors (grade groups 1–3), as well for low-grade tumor modules against modules derived from adjacent non-diseased prostate gland tissue (the “Methods” section). The greatest increase in novelty scores occurred in high-grade primary prostate tumors relative to low-grade tumors (Fig. 4A). In contrast, the novelty scores for modules from low-grade tumors were relatively modest compared to modules from normal tissue samples. Overall, just 23.08% of low-grade modules had medium or high novelty, but 75.96% of high-grade modules had medium or high novelty (Fig. 4B), indicating the majority of changes to co-expression occur during later stages of development of primary tumors in prostate cancer. In both comparisons, mixed UC-MC modules had the highest novelty scores, demonstrating the greatest rewiring in co-expression during prostate cancer progression happens between unicellular and multicellular genes (Fig. 4A). High-grade modules were predominantly mixed UC-MC across all novelty categories (21.31%, 36.61%, and 37.70% high-grade modules were of low, medium, and high novelty modules and mixed UC-MC) were mixed UC-MC across all novelty categories (Fig. 4C).

To understand how co-expression of unicellular and multicellular genes differs between benign and malignant lesions, we analyzed a cohort published by Badal et al. [41], which contains RNA sequencing data from 50 primary melanomas of the skin and 27 benign skin growths known as nevi. We found mixed UC-MC modules from melanomas had significantly higher novelty scores when compared to modules derived from benign nevi than did unicellular-enriched or multicellular-enriched modules (Fig. 4D) (one-sided Wilcoxon test *p*-values = 8.62 × 10^−4^ and 0.059, respectively).

To investigate the full spectrum of co-expression changes during the progression from normal tissue to benign lesions to tumors, we created separate sets of WGCNA modules from RNA sequencing data from benign and malignant pheochromocytoma samples [42], as well as healthy adrenal tissue (the “Methods” section). Tumor samples were further divided into the MAML, SDH, and VHL subtypes, which have distinct driver mutations. Across all subtypes, modules derived from benign tumors showed low novelty as compared to modules from normal adrenal glands, regardless of gene age enrichment. However, novelty scores were markedly higher when comparing modules between malignant and benign pheochromocytomas with mixed UC-MC modules on average having higher novelty than unicellular-enriched and multicellular-enriched modules (Fig. 4E) (Wilcoxon test one-sided *p*-values = 5.88 × 10^−8^, 5.15 × 10^−5^, 9.62 × 10^−15^, comparing mixed UC-MC with UC-enriched in MAML, SDH, and VHL samples, respectively, and 0.0019, 0.10, 1.27 × 10^−13^, comparing mixed UC-MC with MC-enriched in MAML, SDH, and VHL samples, respectively). The melanoma and pheochromocytoma data thus support the notion gene regulatory connections between unicellular and multicellular genes become progressively more altered as tumor cells develop into more malignant states.

### Mixed unicellular-multicellular gene co-expression modules best distinguish low-grade from high-grade glioma tumors

The excess of high-novelty mixed unicellular-multicellular modules in high-grade and malignant tumors indicated the degree of alteration of co-expression of unicellular and multicellular genes distinguishes tumors at different stages of progression. We next hypothesized that these co-expression modules would provide significant discriminant power to classify samples based on their degree of malignancy. To test this, we focused on the low-grade glioma (LGG) and glioblastoma (GBM) datasets of TCGA. LGGs almost inevitably progress to the high-grade and fatal GBM. LGGs and GBMs thus represent linear stages in glioma evolution with clear differences in the degree of malignancy. ssGSEA scores were calculated for the 23 WGCNA modules obtained from the GBM cohort, to measure the combined level of expression of genes within each module in each of the 512 samples from the LGG cohort and the 153 samples from the GBM cohort. Module ssGSEA scores were then used to train an RF model to distinguish between LGG and GBM samples from TCGA (Fig. 4F). RF classification provides a means to determine which co-expression modules are most closely associated with disease stage in glioma. One hundred independent iterations were performed using random splits of training and testing samples each time (the “Methods” section).

The median area under the curve (AUC) of the receiver operating characteristic (ROC) curves for the 100 RF models based on GBM module ssGSEA scores was 0.814 (interquartile range, 0.818–0.894). This was comparable to the performance of RF models trained on the status of mutations normally used to grade adult glioma (median AUC = 0.814), though models based on mutations had much more variable performance (interquartile range, 0.524–0.879) (Additional file 2: Fig. S18). Thus, changes to gene co-expression have the power to accurately classify LGG from GBM samples across patients.

We expected mixed UC-MC modules would be most distinctive between LGG and GBM and therefore carry the greatest weight in the RF classifiers. To investigate, we calculated the mean decrease in the Gini coefficient for each GBM WGCNA module, a measure of the importance of a given variable to an RF model. In total, 11 of the 22 GBM modules had a mean decrease in the Gini coefficient greater than 5, indicating they made a substantial contribution to the predictive power of the RF model (Fig. 4G). Of these, 8 modules were mixed UC-MC with 40–60% genes from multicellular phylostrata (72.7%; Fisher test *p*-value = 0.0296). These results were subsequently validated by random forests trained and tested on orthogonal RNA sequencing data from GBM and LGG patients from the Chinese Glioma Genome Atlas (CGGA). ssGSEA scores for TCGA GBM modules could also distinguish LGG from GBM samples in the CGGA (median AUC = 0.667, IQR 0.640–0.717) (Additional file 2: Fig. S18). In the CGGA RF models, 6 of the 8 modules (75%) with a decrease in Gini > 5 had a mixed UC-MC composition (Additional file 2: Fig. S19). All 6 of these mixed UC-MC modules also had a decrease in Gini > 5 in the TCGA RF models.

The bulk of the predictive power of our gene co-expression-based RF classifiers thus derives from the mixed UC-MC class of WGCNA modules. This underscores the importance of altered co-expression and therefore altered regulatory links between genes of unicellular and multicellular origins in glioma as it progresses from low-grade to high-grade disease. Such changes are biologically meaningful, as distinctive patterns of gene co-expression clearly distinguish LGGs from GBMs in two independent cohorts.

## Discussion

We demonstrate that pronounced alterations to the co-expression of genes of unicellular and multicellular origins are pervasive across tumor transcriptomes. This brings about an imbalance in the co-expression of unicellular and multicellular genes relative to normal, non-malignant tissues. There are a number of important implications for how GRNs that evolved in metazoans to enforce multicellularity are disrupted and rewired in cancer.

First, GRNs get fragmented into smaller and more isolated subnetworks in cancer. More gene co-expression modules were found in tumors, but on average, they contained fewer genes than co-expression modules derived from normal tissues. To quantify the degree of change represented by tumor co-expression modules, we devised a new gene overlap metric termed novelty score. The biggest novelty scores tended to occur in modules in the mixed unicellular-multicellular category. In contrast, tumor modules enriched for unicellular or multicellular genes (UC-enriched or MC-enriched) tended to have low novelty scores. In the context of inferred structures of metazoan GRNs, our analyses suggest major changes occur to GRNs in cancer at the interface between unicellular and multicellular genes to disrupt communication between regions of predominantly unicellular genes or predominantly multicellular genes. This effect likely accompanies and amplifies the dysregulation caused by mutations to GRNs [24].

Mixed UC-MC modules likely have important functional impacts rather than being by-products of transcriptional noise. Mixed UC-MC modules with high novelty scores also showed a trend of higher expression in tumors and a higher prevalence of known driver genes relative to UC-enriched and MC-enriched modules. Somatic mutations may act to force the common expression of pairs of unicellular and multicellular genes that would not normally be co-expressed in healthy somatic tissues [16, 24]. Amplifications in particular can alter the expression of multiple genes simultaneously and thereby rapidly disrupt metazoan GRNs, perhaps explaining their strong enrichment in mixed UC-MC modules. However, many mixed UC-MC modules did not contain known driver genes, indicating environmental, epigenetic, and other factors not explored here cause imbalanced co-expression of unicellular and multicellular genes in tumors as well.

Alterations to co-expression between unicellular and multicellular genes continue as cancer progresses. We observed increased dissimilarity in mixed UC-MC modules from high-grade and malignant tumors relative to their low-grade and benign counterparts, respectively. In contrast, the composition of UC-enriched and MC-enriched modules was comparatively stable across tumor stages. This held across a diverse range of tumors from the prostate, skin, brain, and adrenal gland. We propose the disruption and rewiring of metazoan GRNs are progressive, accelerating at later stages of tumor development to create altered patterns of co-expression of unicellular and multicellular genes that enhance malignancy. Investigation of co-regulatory links between unicellular and multicellular genes may thus uncover new prognostic biomarkers and therapeutic opportunities. The predictive value of mixed UC-MC modules for distinguishing tumor grade in glioma using random forests highlights this potential.

Co-expression analysis carries an inherent degree of uncertainty related to biological and technical variation between samples. While this may impact the accuracy of individual modules, the consistent alteration to the co-expression of unicellular and multicellular genes across 32 diverse types of solid tumors indicates a common and reproducible biological effect across cancers. TCGA samples contain both cancerous cells and non-malignant stroma, and some co-expression modules likely capture features of the tumor microenvironment. However, as mixed UC-MC modules are strongly enriched for processes upregulated in tumors and many are associated with known driver mutations, we expect they largely stem from tumor-intrinsic gene expression changes. Supporting this view, analysis of high-quality single-cell RNA sequencing data from 11 breast cancer patients [46] shows 33/47 (70.2%) of tumor modules from TCGA BRCA cohort are more highly expressed in tumor cells (Additional file 2: Fig. S20). Isolation of tumor cells through single-cell approaches may offer further clarity as techniques for inferring gene regulatory networks de novo from single-cell expression data improve.

The atavism hypothesis of cancer is often interpreted as representing a simple loss of regulation of unicellular genes in cancer. The reality is of course more nuanced, as many genes of multicellular origins are also activated and play key roles in cancer. We propose rather than just suppressing ancient transcriptional programs, metazoan GRNs serve to strike a balance in the co-expression of unicellular and multicellular genes. Integrating activities of core biological programs inherited from unicellular ancestors with novel functionalities and regulatory circuits that emerged with multicellularity enabled the evolution of the stunning array of forms and functions seen in modern metazoans. When regulatory mechanisms enforcing this integration break down, cancer can emerge. Our findings thereby provide a more complex and flexible view of atavism, one in line with recent developments in the field towards a more stepwise and selective process underlying the loss of multicellularity in cancer [11].

## Conclusions

This work creates a foundation for investigating the full implications and mechanisms of how the loss of features of multicellularity drives cancer. Integrating other ‘omics modalities such as proteomic, epigenetic, and chromatin conformation data along with comparative analysis of gene co-expression in unicellular and basal metazoan species with tumors presents fertile ground for future studies.

## Methods

### Phylostratigraphy

We used phylostratigraphy [6] to determine the evolutionary ages of genes, as we have previously done [5]. Briefly, human genes were mapped onto a phylogenetic tree of 16 phylostrata based on sequence similarity, spanning genes found across all organisms (phylostratum 1) to those specific to humans (phylostratum 16). Genes with orthologs in bacteria or single-cell eukaryotes (clades 1–3) were designated as having unicellular origins (UC genes). Genes with orthologs in other multicellular species only were classified as being of multicellular origin (MC genes).

### Gene expression datasets

Tumor and normal gene expression datasets were obtained from The Cancer Genome Atlas (TCGA) for 31 tumor types (adrenocortical carcinoma (ACC), bladder urothelial carcinoma (BLCA), breast invasive carcinoma (BRCA), cervical squamous cell carcinoma and endocervical adenocarcinoma (CESC), cholangiocarcinoma (CHOL), colon adenocarcinoma (COAD), esophageal carcinoma (ESCA), glioblastoma multiforme (GBM), head and neck squamous cell carcinoma (HNSC), kidney chromophobe (KICH), kidney renal clear cell carcinoma (KIRC), kidney renal papillary cell carcinoma (KIRP), brain lower grade glioma (LGG), liver hepatocellular carcinoma (LIHC), lung adenocarcinoma (LUAD), lung squamous cell carcinoma (LUSC), mesothelioma (MESO), ovarian serous cystadenocarcinoma (OV), pancreatic adenocarcinoma (PAAD), pheochromocytoma and paraganglioma (PCPG), prostate adenocarcinoma (PRAD), rectum adenocarcinoma (READ), sarcoma (SARC), skin cutaneous melanoma (SKCM), stomach adenocarcinoma (STAD), testicular germ cell tumors (TGCT), thyroid carcinoma (THCA), thymoma (THYM), uterine corpus endometrial carcinoma (UCEC), uterine carcinosarcoma (UCS), uveal melanoma (UVM)).

For cohorts with data for at least ten normal samples (BLCA, BRCA, COAD, ESCA, HNSC, KICH, KIRC, KIRP, LIHC, LUAD, LUSC, PRAD, READ, STAD, THCA, UCEC), gene expression data from the available matched pairs of tumor-normal samples were used.

To avoid unreliability in the levels of expression of genes, only genes with a count per million (cpm) greater than 1 in either all tumor or all normal samples were included.

### Building gene co-expression modules

WGCNA [30] version 1.61 was used to build gene co-expression modules. As suggested by WGCNA, we used hierarchical clustering based on gene expression to discard dissimilar samples and ensure high consistency between the samples used for the analysis, only keeping paired samples that survived the preprocessing steps. Genes were filtered by WGCNA’s preprocessing function goodSamplesGenes().

Next, the standard procedure to generate gene co-expression modules using WGCNA was followed. Briefly, a large unsigned adjacency matrix was built based on the absolute values of the Pearson correlation coefficients between genes using the adjancency() function. To measure interconnectedness, the topological overlap between genes was calculated using the TOMsimilarity() function. The resulting weighted co-expression network generated by WGCNA represents the genes (nodes) and the strength of co-expression associations between them (edges). Next, hierarchical clustering was performed using hclust() with the “average” agglomerative method, and cutreeDynamic() was used to cut the resulting tree to partition the graph into modules. Modules were required to have at least 30 genes. All modules passed the quality checks performed by WGCNA. The gray module corresponds to genes not assigned to a particular module and was excluded from analyses.

This procedure to build modules was performed independently for each tumor and normal cohort. Besides the membership of genes to modules, we also extracted the interactions between genes in a module by subsetting the initial gene co-expression network built with all genes, resulting in a weighted network of all-against-all genes per module.

### Assigning ages to modules

To assign ages to modules, one-sided Fisher enrichment tests were performed for each module using the number of UC and MC genes in the module and the overall number of UC and MC genes in the gene expression cohort dataset used to generate the modules. Correction for multiple testing was performed using the Benjamini–Hochberg procedure, and significance was achieved when the adjusted *p*-value was less than 0.05. Modules were classified as UC if they were enriched in UC genes, MC if they were enriched in MC genes, and mixed UC-MC if they were not enriched in either.

### Functional enrichment of modules

We used the gProfiler [33] R package to perform functional enrichment of modules using gene sets from the Kyoto Encyclopedia of Genes and Genomes [31] and Reactome [32] using the g:GOst function, which performs over-representation analysis. The top functional enrichment categories were selected for each module. Pathways unrelated to cancer and general cancer pathways were excluded. Specifically, we excluded the following terms: Huntington disease, human cytomegalovirus infection, Alzheimer disease, *Staphylococcus aureus* infection, central carbon metabolism in cancer, hepatitis C, microRNAs in cancer, proteoglycans in cancer, measles, pertussis, small cell lung cancer, hepatocellular carcinoma, viral myocarditis, Parkinson disease, insulin resistance, human papillomavirus infection, diseases of signal transduction, chronic myeloid leukemia, HIV infection, pathways in cancer, loss of function of SMAD4 in cancer, *Vibrio cholerae* infection, platinum drug resistance, hypertrophic cardiomyopathy (HCM), gastric cancer, non-small cell lung cancer, toxoplasmosis, Epstein-Barr virus infection, renal cell carcinoma, shigellosis, basal cell carcinoma, colorectal cancer, endometrial cancer, human immunodeficiency virus 1 infection, Kaposi sarcoma-associated herpesvirus infection, herpes simplex virus 1 infection, AGE-RAGE signaling pathway in diabetic complications, defective CFTR causes cystic fibrosis, dilated cardiomyopathy (DCM), hepatitis B, transcriptional misregulation in cancer, non-alcoholic fatty liver disease (NAFLD), infectious disease, Chagas disease (American trypanosomiasis), signaling by FGFR1 in disease, and SMAD4 MH2 domain mutants in cancer. Modules without functional enrichment were excluded.

### Novelty scores of modules

Intuitively, the novelty score of a tumor module corresponds to how much of the module is found in normal modules.

First, we defined the raw novelty of a tumor module *A* as the minimum number (*N*) of normal modules that share 50% of the genes of the tumor module *A*. To calculate this, normal modules derived from the matched normal tissue are sorted by decreasing order of the number of genes in common with the tumor module. Then, starting from the module with the largest overlap, we count the number of normal modules that would be needed to achieve the 50% cutoff. This number represents the raw novelty score. The higher the novelty score, the greater the number of normal modules needed to achieve the cutoff, and therefore, the tumor module is more specific to tumor samples.

To normalize the association between novelty and the number of genes in the tumor module, we calculated the ratio between the raw novelty score of a module and the module size (*S*) (Eq. 1).

1 $${{\text{Novelty}}}_{A}=\frac{{{\text{Rawnovelty}}}_{A}}{{S}_{A}}$$

Modules were subsequently classified as being of high, moderate, or low novelty by using the 1/3 and 2/3 quantiles as cutoffs. As a result, “high novelty” modules are the most tumor-specific, and “low novelty” modules are modules that are largely also found in normal samples.

### Level of expression of modules

The level of expression of modules was determined using ssGSEA [43] from the GSVA package [34] where genes of each sample are ranked based on their levels of expression. Higher scores are given when the genes of a module are consistently highly ranked.

### Somatic mutation analysis

A list of cancer drivers was obtained from the COSMIC Cancer Census database (version Dec 21 23_46_30 2022) [44]. Genes were classified based on the disease where they have been reported to the drivers as per COSMIC and were used in the analyses of the corresponding tumor type. Modules without cancer census genes were excluded from the analysis.

Point mutation and copy number data for the tumor samples were obtained from TCGA and filtered as we have done previously [24]. A gene was considered to be recurrently point mutated if it had a missense or LoF mutation in at least three patients and a non-synonymous to synonymous ratio of less than 1. A gene was considered recurrently amplified or deleted if this occurred in at least 10% of the samples in the individual tumor cohort. Only focal amplifications and deletions were considered. We classified a gene as being focally amplified or deleted genes if its copy number segment was in the upper quantile (0.25) of the distribution of the mean fraction of copy number aberrant chromosomes across patients for each chromosome.

### Gene centrality

To calculate gene centrality, we first calculated the degree of genes in the co-expression networks, which was calculated as the sum of the weights of the gene with all other genes in the network. To account for the differences in the number of genes and overall strength of modules, the degree values were then normalized by dividing by the median degree of the module. To ensure the values were comparable across tumor types, genes were ranked by their normalized degree values. Subsequently, the ranks were divided by the size of the module, resulting in “relative” ranks, with the maximum value being 1. A value of 1 was subtracted from the resulting values. Higher values would represent genes with a higher centrality, and lower values those that were more peripheral.

### Prostate cancer dataset

The RNAseq prostate adenocarcinoma dataset was obtained from TCGA as described above. Tumor samples were further classified based on pathological annotations. Samples belonging to grade groups 1–3 (Gleason patterns 2 + 4, 3 + 3, 3 + 4, 4 + 3) were deemed to be “low grade,” and those of grade groups 4–5 (Gleason patterns 4 + 4, 3 + 5, 5 + 3, 5 + 4, 4 + 5, 5 + 5) were classified as “high grade.” This resulted in 45 low-grade samples and 7 high-grade samples, each with a matched normal sample.

Gene co-expression modules were derived from each sample set independently. Novelty scores were calculated comparing modules from low-grade samples and those from normal samples, as well as from high-grade samples to low-grade samples. Modules were classified as high, medium, or low novelty as described above using both sets of modules.

### Benign skin nevi and melanoma dataset

Gene expression data was obtained from Badal et al. [41] and downloaded from https://www.ncbi.nlm.nih.gov/geo/query/acc.cgi?acc=GSE98394. This dataset included 27 nevi samples and 51 primary tumor samples; however, 1 tumor sample was excluded during pre-processing as it was an outlier. Gene co-expression modules were derived from each sample set independently. Novelty scores were calculated by comparing modules from primary and nevi samples. Modules were classified as high, medium, or low novelty as described above.

### Benign and malignant pheochromocytomas

Gene expression data was obtained from previously published work [42]. This dataset included 17 normal samples from healthy adrenal tissue, as well benign and malignant samples from 3 subtypes of pheochromocytomas: MAML (9 benign and 8 malignant samples), SDH (44 benign and 36 malignant samples), and VHL (77 benign and 8 malignant samples).

Gene co-expression modules were derived from each sample set independently. Novelty scores were calculated by comparing modules from benign and normal samples, as well as malignant and benign samples. Modules were classified as high, medium, or low novelty as described above.

### Random forest models

Single-sample GSEA scores (ssGSEA) for the 23 WGCNA modules derived from the TCGA glioblastoma (GBM) cohort were calculated for each RSEM-normalized RNA sequencing sample from the TCGA GBM and low-grade glioma (LGG) cohorts, using the GSVA package as described above. Random forest models were trained on ssGSEA scores and tested for their accuracy in the classification of LGG and GBM samples. A 70/30 split was used for training and testing, repeated for 100 random splits of samples for training and testing. The area under the curve (AUC) for the receiver operating characteristic (ROC) was recorded to assess model accuracy, and the mean decrease in Gini coefficients for each module was recorded to measure variable importance. For validation, the mRNAseq 693 (batch 1) RSEM RNA sequencing gene expression dataset was downloaded from the Chinese Glioma Genome Atlas (CGGA) (http://www.cgga.org.cn/index.jsp) [45]. The dataset contained 693 samples of both primary and recurrent low-grade gliomas and glioblastomas. Random forest model training and testing for accuracy in the classification of LGG and GBM samples were conducted as described for TCGA data. Model training and performance assessment were performed in R 3.6.3 using the randomForest (v4.6–14) and pROC (v1.17.0.1) packages.

#### Generation of random modules

We randomly assigned genes to modules keeping the same module sizes. This was repeated 1000 times. The age enrichment was previously described for each iteration.

#### Generation of modules with the strongest connections

We selected the top 50% strongest edge weights from each module. This resulted in genes being connected to only a subset of other genes in the module, resulting in fragmentation of the module. To minimize this effect, we performed a second filtering step, where we only kept genes that were connected to at least half of the genes in the module. Next, gene age enrichment and novelty analyses were performed as previously described.

#### Calculation of module overlaps

To evaluate the degree to which UC-MC modules were shared across tumor types, we calculated the percentage of gene overlap between modules from each TCGA cohort. For each module, we recorded the percentage overlap for the module with which it had the maximum overlap in each cohort. To summarize the results, we calculated the average overlap per module for each cancer type.

#### Module expression in single-cell data

Data from single-cell sequencing of 11 breast tumors (GSE176078) [46] was processed using Seurat with standard settings. Tumor cells were identified based on the expression of EPCAM. The remaining cells were deemed normal (non-malignant) cells. Only samples with at least 500 tumor cells were selected, resulting in 11 samples. Module score of each breast cancer tumor module in each sample was calculated using the AddModuleScore() function. We then calculated the difference in scores between tumor and normal cells. One-sided Wilcoxon rank-sum tests were applied to compare scores between tumor and normal cells, followed by correction for multiple testing using Benjamini–Hochberg correction. Adjusted *p*-values < 0.01 were considered significant. Modules with significantly higher scores in tumor cells in 6 or more patient samples (i.e., more than half of the samples) were considered to have higher overall expression in tumor cells.

### Supplementary Information

**Additional file 1: Dataset S1.** Phylostrata assignments of human genes.

**Additional file 2: Fig. S1.** Number of co-expression modules for cohorts with only matched tumour and normal samples. **Fig. S2.** Correlation between number of modules/modules sizes and cohort size. Spearman correlation values of top panel: Normal= -0.32, Tumour = -0.12. Spearman correlation values of bottom panel: Normal = -0.0059, Tumour = -0.53. **Fig. S3.** Module sizes for cohorts with only matched tumour and normal samples. **Fig. S4.** Percentage of genes assigned to modules per cohort. The percentage of unassigned genes correspond to those in the ‘grey’ module. **Fig. S5. **Fraction of age enrichment categories among tumour and normal modules. **Fig. S6.** Module properties after removal of connections below the median edge weight for each module. (A) Number of modules in each age enrichment category, for Normal and Tumour sample cohorts. (B) Novelty scores for Tumour modules in each age enrichment category. **Fig. S7.** Overlap in gene content between co-expression modules. (A, B) Example heatmaps showing overlap between modules from the CESC cohort against all others of the TCGA cohort (A) and against randomized modules (B). Colours represent similarity score for best-matching modules in each of the other TCGA cohorts. (C, D) Summary density plot of the mean overlap of modules of each cohort with the other TCGA cohorts and with the randomized modules from tumour (C) and normal (D) cohorts. The distribution of module overlap scores between TCGA cohorts is shifted to higher higher values than overlap distribution for modules generated by random selection of genes. **Fig. S8.** Differences in Novelty scores for UC-enriched, Mixed UC-MC and MC-enriched tumours across all cohorts. **Fig. S9.** Fraction of modules of each age per novelty category in each tumour cohort. **Fig. S10.** Percentage of connections that are UC-MC in Mixed UC-MC, UC-enriched and MC-enriched modules. **Fig. S11.** Proportions of high, medium and low Novelty modules with ssGSEA scores in the upper quartiles of all ssGSEA scores calculated for their respective tumour cohorts. **Fig. S12.** Correlation between module novelty score and percentage of known cancer driver genes from COSMIC Cancer Census gene list, stratified by module novelty. **Fig. S13. **Normalized degree of recurrently amplified genes within WGCNA co-expression modules from normal (left) and tumour (right) cohorts, coloured according to tumour type. **Fig. S14.** Changes in centrality for driver genes in other solid tumour cohorts from TGCA. **Fig. S15.** Centrality of recurrently amplified and non-mutated genes in modules from tumour cohorts. **Fig. S16.** Centrality of recurrently deleted and non-mutated genes in modules from tumour cohorts. **Fig. S17.** Survival curves for low vs high expression of LGG purple module. **Fig. S18.** AUCs of Random Forest models trained on mutation status from TCGA (green), ssGSEA scores of GBM modules tested on CGGA (red) and TCGA (blue) datasets. **Fig. S19.** Mean decrease in Gini Coefficient for GBM modules from TCGA from 100 iterations of Random Forest models tested on RNA-seq data from the CGGA. **Fig. S20.** Expression of WGCNA modules from tumour samples derived from TCGA BRCA cohort in cells of breast tumours and surrounding non-tumour cells of the stroma profiled by single-cell RNA sequencing from Wu et al (GSE176078). Representative examples of a BRCA tumour module (A) expressed predominantly in tumour cells, (B) with broad expression across cell types, or (B) with expression mostly restricted to stromal cells. (D) Difference in expression of BRCA tumour modules in tumour as compared to normal cells for UC-enriched (red), Mixed UC-MC (green) and MC-Enriched (blue) age enrichment categories. (E) Number of BRCA tumour modules with higher expression in tumor cells (dark grey) or normal cells (light grey), as defined by having significantly higher gene set scores in 6 out of the 11 patients included in the dataset. **Table S1.** Number of modules for Tumours & Normal cohorts by age enrichment category.

**Additional file 3.** Review history.

## Data Availability

Tumor and normal gene expression datasets for the 31 tumor types included in this work are available from The Cancer Genome Atlas (TCGA) [47]. Nevi and melanoma datasets are available at the NCBI GEO database (https://www.ncbi.nlm.nih.gov/geo/query/acc.cgi?acc=GSE98394) [48]. The pheochromocytoma data is available from previously published work [42]. Data from the Chinese Glioma Genome Atlas is available at http://www.cgga.org.cn/index.jsp [49]. The list of genes assigned to modules in each cancer type and the code to reproduce all analyses is available at https://github.com/cancer-evolution/Evolutionary_analysis_of_coexpression_modules (10.5281/zenodo.10929865) under the GPL-3.0 license [50, 51].
