# Supplementary material for: Disruption of metazoan gene regulatory networks in cancer alters the balance of co-expression between genes of unicellular and multicellular origins
Source: Genome Biol. 2024 Apr 29;25:110. doi: 10.1186/s13059-024-03247-1 (PMC11057133; doi:10.1186/s13059-024-03247-1)
Supplement: Supplementary file 2 — Additional file 2: Fig. S1. Number of co-expression modules for cohorts with only matched tumour and normal samples. Fig. S2. Correlation between number of modules/modules sizes and cohort size. Spearman correlation values of top panel: Normal= -0.32, Tumour = -0.12. Spearman correlation values of bottom panel: Normal = -0.0059, Tumour = -0.53. Fig. S3. Module sizes for cohorts with only matched tumour and normal samples. Fig. S4. Percentage of genes assigned to modules per cohort. The percentage of unassigned genes correspond to those in the ‘grey’ module. Fig. S5. Fraction of age enrichment categories among tumour and normal modules. Fig. S6. Module properties after removal of connections below the median edge weight for each module. (A) Number of modules in each age enrichment category, for Normal and Tumour sample cohorts. (B) Novelty scores for Tumour modules in each age enrichment category. Fig. S7. Overlap in gene content between co-expression modules. (A, B) Example heatmaps showing overlap between modules from the CESC cohort against all others of the TCGA cohort (A) and against randomized modules (B). Colours represent similarity score for best-matching modules in each of the other TCGA cohorts. (C, D) Summary density plot of the mean overlap of modules of each cohort with the other TCGA cohorts and with the randomized modules from tumour (C) and normal (D) cohorts. The distribution of module overlap scores between TCGA cohorts is shifted to higher higher values than overlap distribution for modules generated by random selection of genes. Fig. S8. Differences in Novelty scores for UC-enriched, Mixed UC-MC and MC-enriched tumours across all cohorts. Fig. S9. Fraction of modules of each age per novelty category in each tumour cohort. Fig. S10. Percentage of connections that are UC-MC in Mixed UC-MC, UC-enriched and MC-enriched modules. Fig. S11. Proportions of high, medium and low Novelty modules with ssGSEA scores in the upper quartiles of all ssGSEA scores [file 13059_2024_3247_MOESM2_ESM.docx]

**Supporting Information for**

Disruption of metazoan gene regulatory networks in cancer alters the balance of co-expression between genes of unicellular and multicellular origins

Anna S. Trigos^1,2*^, Felicia Bongiovanni^1,2^, Magnus Zethoven^1,2^, Richard Tothill^3^, Richard B. Pearson^1,2,4,5^, Tony Papenfuss^1,2,6^, David L. Goode^1,2*^

Corresponding Authors: David L. Goode, Anna S. Trigos

Email: [david.goode@petermac.org](mailto:david.goode@petermac.org), [anna.trigos@petermac.org](mailto:anna.trigos@petermac.org)

**This PDF file includes:**

Figures S1 to S20

Table S1

**Other supporting materials for this manuscript include the following:**

Dataset S1 (Additional file 1)

Fig S1. Number of co-expression modules for cohorts with only matched tumour and normal samples.

Fig S2. Correlation between number of modules/modules sizes and cohort size. Spearman correlation values of top panel: Normal= -0.32, Tumour = -0.12. Spearman correlation values of bottom panel: Normal = -0.0059, Tumour = -0.53.

Fig S3. Module sizes for cohorts with only matched tumour and normal samples.

Fig S4. Percentage of genes assigned to modules per cohort. The percentage of unassigned genes correspond to those in the ‘grey’ module.

Fig S5. Fraction of age enrichment categories among tumour and normal modules.

**A**

**B**

**Fig S6.** Module properties after removal of connections below the median edge weight for each module. (A) Number of modules in each age enrichment category, for Normal and Tumour sample cohorts. (B) Novelty scores for Tumour modules in each age enrichment category.

Fig S7: Overlap in gene content between co-expression modules. (A, B) Example heatmaps showing overlap between modules from the CESC cohort against all others of the TCGA cohort (A) and against randomized modules (B). Colours represent similarity score for best-matching modules in each of the other TCGA cohorts. (C, D) Summary density plot of the mean overlap of modules of each cohort with the other TCGA cohorts and with the randomized modules from tumour (C) and normal (D) cohorts. The distribution of module overlap scores between TCGA cohorts is shifted to higher higher values than overlap distribution for modules generated by random selection of genes.

Fig S8. Differences in Novelty scores for UC-enriched, Mixed UC-MC and MC-enriched tumours across all cohorts.

Fig S9. Fraction of modules of each age per novelty category in each tumour cohort.

Fig S10. Percentage of connections that are UC-MC in Mixed UC-MC, UC-enriched and MC-enriched modules.

Fig S11. Proportions of high, medium and low Novelty modules with ssGSEA scores in the upper quartiles of all ssGSEA scores calculated for their respective tumour cohorts.

Fig S12. Correlation between module novelty score and percentage of known cancer driver genes from COSMIC Cancer Census gene list, stratified by module novelty.

Fig S13. Normalized degree of recurrently amplified genes within WGCNA co-expression modules from normal (left) and tumour (right) cohorts, coloured according to tumour type.

Fig S14. Changes in centrality for driver genes in other solid tumour cohorts from TGCA.

Fig S15. Centrality of recurrently amplified and non-mutated genes in modules from tumour cohorts.

Fig S16. Centrality of recurrently deleted and non-mutated genes in modules from tumour cohorts.


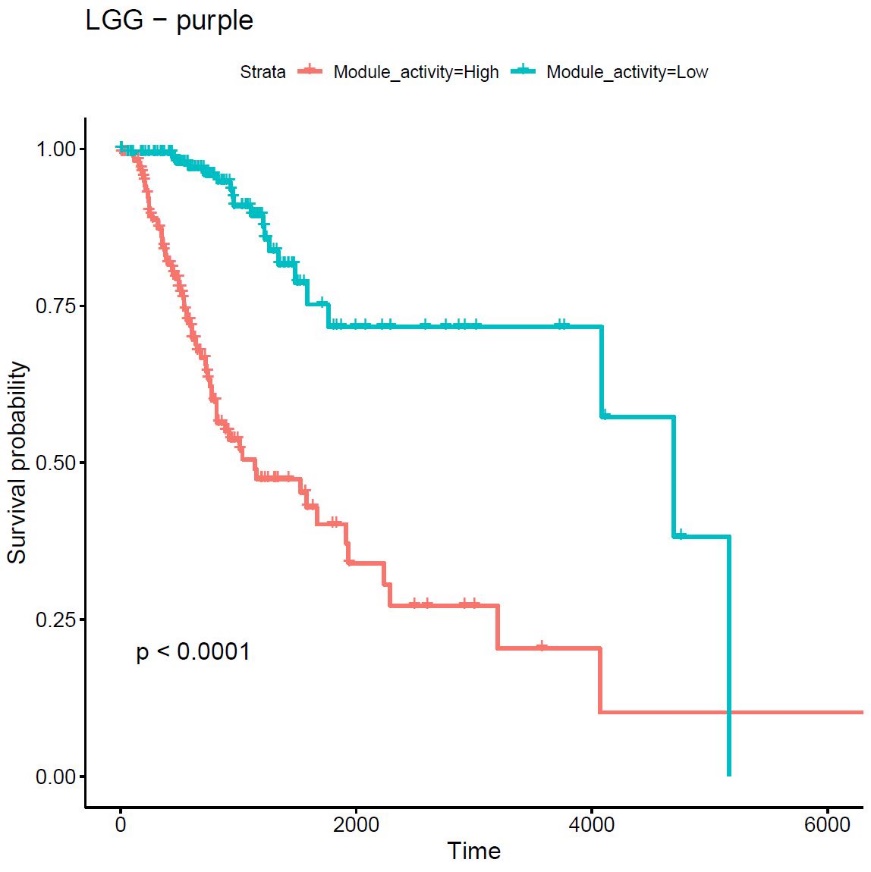


Fig S17. Survival curves for low vs high expression of LGG purple module.


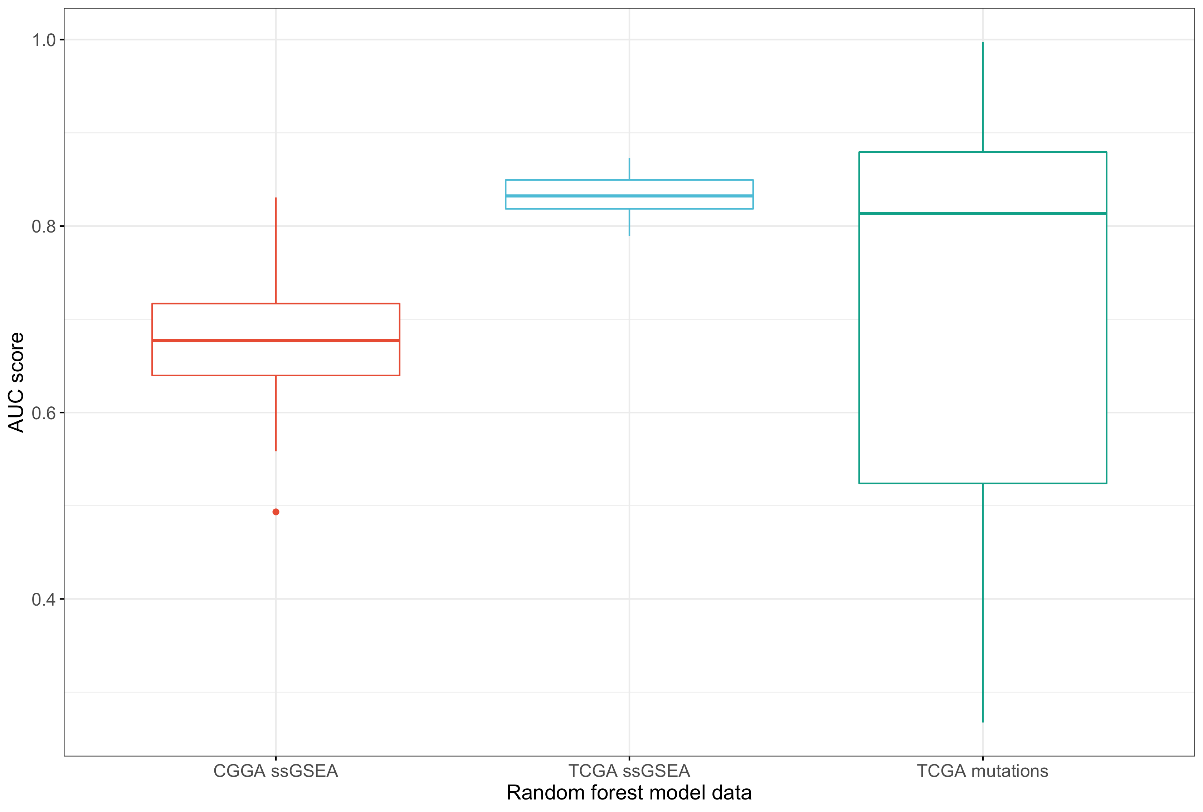


Fig S18. AUCs of Random Forest models trained on mutation status from TCGA (green), ssGSEA scores of GBM modules tested on CGGA (red) and TCGA (blue) datasets.


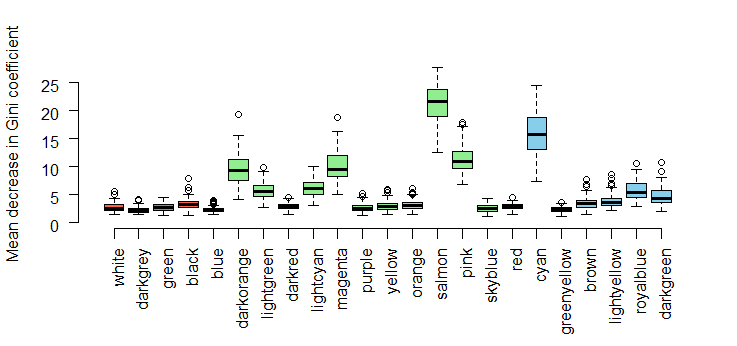


Fig S19. Mean decrease in Gini Coefficient for GBM modules from TCGA from 100 iterations of Random Forest models tested on RNA-seq data from the CGGA.


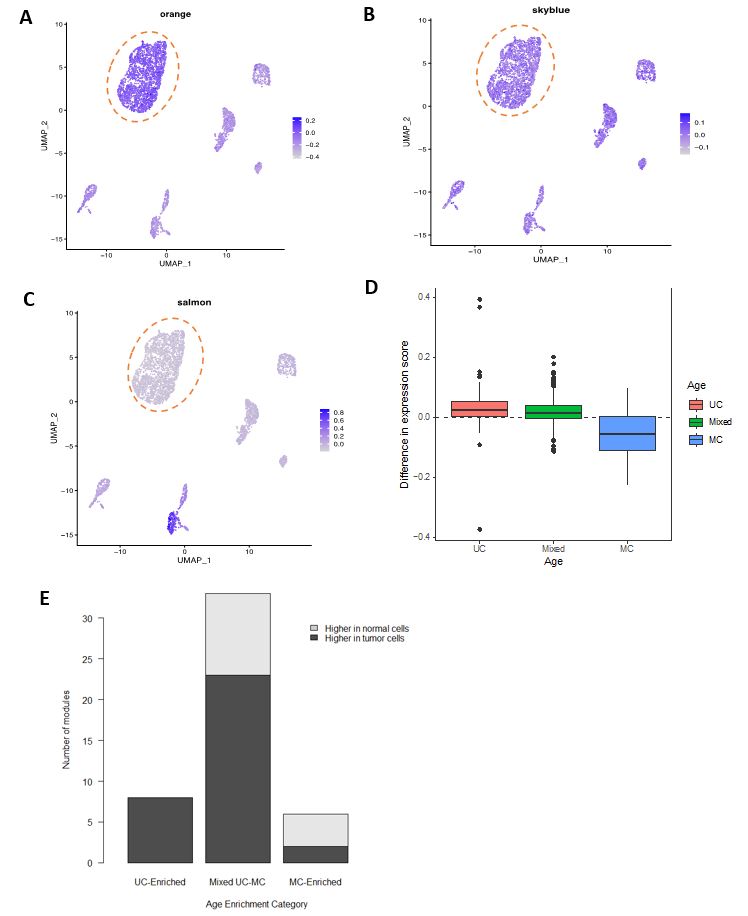
Fig S20. Expression of WGCNA modules from tumour samples derived from TCGA BRCA cohort in cells of breast tumours and surrounding non-tumour cells of the stroma profiled by single-cell RNA sequencing from Wu et al (GSE176078). Representative examples of a BRCA tumour module (A) expressed predominantly in tumour cells, (B) with broad expression across cell types, or (B) with expression mostly restricted to stromal cells. (D) Difference in expression of BRCA tumour modules in tumour as compared to normal cells for UC-enriched (red), Mixed UC-MC (green) and MC-Enriched (blue) age enrichment categories. (E) Number of BRCA tumour modules with higher expression in tumor cells (dark grey) or normal cells (light grey), as defined by having significantly higher gene set scores in 6 out of the 11 patients included in the dataset.

Table S1: Number of modules for Tumours & Normal cohorts by age enrichment category.

|  |  | |  | |  | **Number of Co-expression Modules** | | | | |  | |  |
| --- | --- | --- | --- | --- | --- | --- | --- | --- | --- | --- | --- | --- | --- |
| **Module Age Enrichment Category** | | **Sample Type** | | **Minimum** | | | **1st Quartile** | **Median** | **Mean** | **3rd Quartile** | | **Maximum** | **Total** |
| Unicellular-enriched | | Tumour | | 1 | | | 4 | 6 | 5.97 | 8 | | 12 | 185 |
|  | | Normal | | 1 | | | 3.75 | 5.5 | 5.25 | 7 | | 8 | 84 |
| Mixed Unicellular-Multicellular | | Tumour | | 6 | | | 14.5 | 20 | 24.97 | 31 | | 82 | 774 |
|  | | Normal | | 2 | | | 6 | 9 | 8.56 | 11 | | 16 | 137 |
| Multicellular-enriched | | Tumour | | 1 | | | 6 | 7 | 6.67 | 8 | | 10 | 207 |
|  | | Normal | | 2 | | | 6.75 | 7 | 7.25 | 8 | | 13 | 116 |
