# Supplementary material for: Disruption of metazoan gene regulatory networks in cancer alters the balance of co-expression between genes of unicellular and multicellular origins
Source: Genome Biol. 2024 Apr 29;25:110. doi: 10.1186/s13059-024-03247-1 (PMC11057133; doi:10.1186/s13059-024-03247-1)
Supplement: Supplementary file 3 — Additional file 3. Review history. [file 13059_2024_3247_MOESM3_ESM.docx]

Review History

**First round of review**

**Reviewer 1**

**Are you able to assess all statistics in the manuscript, including the appropriateness of statistical tests used?** Yes, and I have assessed the statistics in my report.

**Comments to author:**

In this manuscript the authors attempt to find further evidence for the atavism hypothesis of cancer by looking at the gene co-expression modules. They found in cancer samples many new modules composed of both unicellular (UC) and multicellular (MC) genes. Such UC-MC modules are enriched with known cancer drivers and more frequent in advanced tumors. The manuscript is generally clear, but there are several issues to be clarified before acceptance.

First, the co-expression modules obtained by WGCNA are not necessarily co-expressed; WGCNA tends to cluster as many input genes as possible into modules even they are not that co-expressed. Because in normal samples UC genes and MC genes tend to be in distinct co-expression modules, it is expected that random changes of gene expressions would produce new UC-MC modules. Hence, it would be ideal to test if the observed UC-MC modules result from random gene expression changes. The authors may include a simulation to address this issue

Second, the enrichment of cancer drivers in UC-MC modules supports the functional importance of these modules in cancer. However, I would expect some co-occurrences across cancer types for the UN-MC modules.

Third, as the authors mentioned in discussion, the many types of normal cells in a tumor would complicate the expression analysis. Given the availability of sc-RNA data of many tumors, the authors can easily make a stronger argument by including those sc-RNA data.

**Reviewer 2**

**Are you able to assess all statistics in the manuscript, including the appropriateness of statistical tests used?** Yes, and I have assessed the statistics in my report.

**Comments to author:**

Anna S Trigos et al present an interesting work to show that the disruption of metazoan gene regulatory networks in cancer alters the balance of co-expression between genes of unicellular and multicellular origins. They found the greatest differences between tumour and normal tissue co-expression networks occurring within Mixed UC-MC modules. MC and UC genes not commonly co-expressed in normal tissues formed distinct co-expression modules seen only in tumours. The degree of rewiring of genes within Mixed UC-MC modules increased with tumour grade and stage. Mixed UC-MC modules were enriched for somatic mutations in cancer genes, particularly amplifications, suggesting an important driver of the rewiring observed in tumours are copy number changes. The authors concluded that disruption and rewiring of metazoan GRNs is a common and important aspect of tumour progression that causes imbalance in the co-expression of unicellular and multicellular genes.

This is a well-conducted study, and the findings are generally convincing, and should be of broad interests to the researchers in the field. I have only two minor points:

1) The authors showed an striking pattern on the gene co-expression between genes with different ages, i.e., of unicellular and multicellular origins. Was there specific reason to classify the gene age into these two groups? And also, could more detailed step-wise pattern can be observed, if the gene ages can be classified into more groups, e.g., mammalian specific genes, or primate specific genes?

2) The supplementary Data can not be found with the submission, and git hub repository link does not work.

**Authors Response**

**Point-by-point responses to the reviewers’ comments:**

Reviewer #1: In this manuscript the authors attempt to find further evidence for the atavism hypothesis of cancer by looking at the gene co-expression modules. They found in cancer samples many new modules composed of both unicellular (UC) and multicellular (MC) genes. Such UC-MC modules are enriched with known cancer drivers and more frequent in advanced tumors. The manuscript is generally clear, but there are several issues to be clarified before acceptance.

First, the co-expression modules obtained by WGCNA are not necessarily co-expressed; WGCNA tends to cluster as many input genes as possible into modules even they are not that co-expressed. Because in normal samples UC genes and MC genes tend to be in distinct co-expression modules, it is expected that random changes of gene expressions would produce new UC-MC modules. Hence, it would be ideal to test if the observed UC-MC modules result from random gene expression changes. The authors may include a simulation to address this issue.

While it is true co-expression analysis is sensitive to random variability in gene expression, we now provide 3 lines of evidence that UC-MC modules identified by WGCNA in tumours result from genuine patterns to co-expression representative of gene regulatory changes that are consistent across cancers.

1) Randomly assigning genes to modules within each tumour type resulted in very different patterns of gene age enrichment than were observed in TCGA tumour data. Nearly all random modules contained a mix of UC and MC genes (Figure R1), in contrast to actual modules which have UC-enriched, MC-enriched and Mixed UC-MC types in all cohorts. These results have been added to the Supplementary Materials as Fig S6, with details of the analysis added to the Methods section.

This aligns with our hypothesis that human gene regulatory networks (GRNs) are composed of regions that are predominantly unicellular or multicellular, with interface regions containing genes of both types, whereas random expression patterns would not show such structure. The mixed UC-MC regions should change the most in cancers but certain UC-enriched and MC-enriched regions would remain given their general importance to cell growth and survival. The resulting GRN structure should be maintained in tumours, which is indeed what we observe.

**Figure R1**: Proportion of observed Normal, observed Tumour and randomly generated modules that are UC-enriched, MC-enriched or Mixed UC-MC.

2) When weaker connections are removed from WGCNA modules, i.e., ones more likely to represent random ‘noise’, the overall patterns of gene age enrichment are maintained. Figure R2 below (now included as Supp Fig S6) demonstrates the results closely match the original analysis presented in Figures 1D and 2C after gene-gene connections with edge weights below the median (i.e., bottom 50%) are removed. Mixed UC-MC modules are still over-represented in tumours (Panel A), and Novelty is still highest for Mixed UC-MC modules (Panel B).

**A**

**B**

**Figure R2**: Module properties after removal of connections below the median edge weight for each module. (A) Number of modules in each age enrichment category, for Normal and Tumour sample cohorts. (B) Novelty scores for Tumour modules in each age enrichment category.

3) Pertaining to Reviewer 1’s next comment, the overlap between genes across different sets of randomly assigned modules is consistently lower than the overlap seen between Mixed UC-MC modules from different tumour cohorts (Figure R3), indicating most Mixed modules are not solely due to random expression.

Second, the enrichment of cancer drivers in UC-MC modules supports the functional importance of these modules in cancer. However, I would expect some co-occurrences across cancer types for the UN-MC modules.

To evaluate the degree to which UC-MC modules were shared across tumour types, we calculated the percentage of gene overlap between modules from each TCGA cohort. For each module, we recorded the percentage overlap for the module with which it had the maximum overlap in each cohort (Fig R3A shows an example overlap heatmap for the CESC cohort). When comparing to random modules (Fig R3B), the level of overlap between modules was much lower. To compare across cohorts, we took the mean of these maximum overlap scores. Modules with high similarity in many cohorts got a high score, while modules that were unique to one cancer type would get a low score.

As shown in Figure R3C, scores for observed UC-MC modules were higher than the random modules we generated (see above) and, importantly, there was a long tail of modules with ‘high’ similarity across cancer types. This indicates a substantial proportion of Mixed UC-MC modules share genes across cohorts and are therefore likely represent recurrent changes in co-expression. This is also the case for modules from normal tissue samples (Fig R3B and R3D).

Figure R3 is now included the Supplementary Materials, as Figure S7. The full set of module overlap heatmaps have been added to the GitHub repository.

It is worth reiterating several KEGG and Reactome terms were found to be recurrently enriched in Mixed UC-MC across multiple cancer types (Fig 1E), as alluded to by the reviewer.

**Figure R3**: Overlap in gene content between co-expression modules. (A, B) Example heatmaps showing overlap between modules from the CESC cohort against all others of the TCGA cohort (A) and against randomized modules (B). Colours represent similarity score for best-matching modules in each of the other TCGA cohorts. (C, D) Summary density plot of the mean overlap of modules of each cohort with the other TCGA cohorts and with the randomized modules from tumour (C) and normal (D) cohorts. The distribution of module overlap scores between TCGA cohorts is shifted to higher values than the overlap distribution for modules generated by random selection of genes.

Third, as the authors mentioned in discussion, the many types of normal cells in a tumor would complicate the expression analysis. Given the availability of sc-RNA data of many tumors, the authors can easily make a stronger argument by including those sc-RNA data.

True, a variety of scRNA-seq data now available to investigate the contribution of normal cells in the tumour microenvironment (TME) to our analysis. It would be quite an undertaking to download, align, process and normalize data from all 31 tumour types included in our manuscript, however.

To complete our analysis in a workable timeframe, we chose a large single-cell breast cancer data sets with readily available expression matrices (GSE176078) published by Wu et al in *Nature Genetics* in 2021 (PMID 35933466). We feel this is sufficient to get a sense of the relative expression of our modules within tumour cells and the adjacent TME.

Data were processed and clustered using Seurat, and relevant marker genes used to identify tumour and normal cells in each sample. While a few modules in each tumour type were found predominantly in cells of the TME (Fig R4C), the majority of co-expression modules identified from the matching TCGA cancer cohort were expressed in tumour cells in the scRNA-seq data (Fig R4A/B). Although many cancer modules were also expressed by normal cells, the expression was seen across multiple cell types (Fig R4B), indicating cancer modules are unlikely to simply reflect differences in TME composition.

To better align to the bulk RNA-seq analysis we performed for TCGA data, we calculated a score for the expression of each module across cells. UC-Enriched and Mixed UC-MC Cancer modules showed higher expression in tumour cells versus normal cells (Fig R4D), indicating stronger contribution of tumour cells over cells of the TME in the formation of this class of co-expression modules. As expected, MC-enriched modules on average had showed higher expression in stromal cells.

Overall, all 8 of the UC-enriched modules were on average more highly expressed in tumour cells, while the majority (23/33) of Mixed UC-MC modules also had higher average expression in tumour cells (Fig R4E).

While it may seem unexpected to have genes in tumour modules expressed in normal cells, many genes expressed in tumour are also expressed in normal cells. The Seurat methodology will detect transcription of those genes even when they are not strongly co-expressed as they are in tumour cells.

Figure R4 has been added to the Supplementary Materials, as Supplementary Figure S20.

**Figure R4**: Expression of co-expression modules detected by WGCNA in tumour samples from TCGA BRCA cohort in cells of breast tumours and surrounding non-tumour cells of the stroma profiled by single-cell RNA sequencing from Wu et al (GSE176078). Representative examples of a BRCA tumour module (A) expressed predominantly in tumour cells, (B) with broad expression across cell types, or (B) with expression mostly restricted to stromal cells. (D) Difference in expression of BRCA tumour modules in tumour as compared to normal cells for UC-enriched (red), Mixed UC-MC (green) and MC-Enriched (blue) age enrichment categories. (E) Number of BRCA tumour modules with higher expression in tumor cells (dark grey) or normal cells (light grey), as defined by having significantly higher gene set scores in 6 out of the 11 patients included in the dataset.

Reviewer #2: Anna S Trigos et al present an interesting work to show that the disruption of metazoan gene regulatory networks in cancer alters the balance of co-expression between genes of unicellular and multicellular origins. They found the greatest differences between tumour and normal tissue co-expression networks occurring within Mixed UC-MC modules. MC and UC genes not commonly co-expressed in normal tissues formed distinct co-expression modules seen only in tumours. The degree of rewiring of genes within Mixed UC-MC modules increased with tumour grade and stage. Mixed UC-MC modules were enriched for somatic mutations in cancer genes, particularly amplifications, suggesting an important driver of the rewiring observed in tumours are copy number changes. The authors concluded that disruption and rewiring of metazoan GRNs is a common and important aspect of tumour progression that causes imbalance in the co-expression of unicellular and multicellular genes.

This is a well-conducted study, and the findings are generally convincing, and should be of broad interests to the researchers in the field. I have only two minor points:

1) The authors showed an striking pattern on the gene co-expression between genes with different ages, i.e., of unicellular and multicellular origins. Was there specific reason to classify the gene age into these two groups? And also, could more detailed step-wise pattern can be observed, if the gene ages can be classified into more groups, e.g., mammalian specific genes, or primate specific genes?

We appreciate the supportive comments and thought-provoking questions from Reviewer 2.

In response to their first question, since our main interest is on the general, broad loss of features of multicellularity in cancer, rather than particular features of multicellularity, we chose to group genes from all multicellular phylostrata together and compare them to all unicellular phylostrata. We consider this to be the most important and relevant evolutionary boundary because it gave rise to the coordination and cooperation between cells that remained highly conserved in metazoans but get subverted in cancer.

Step-wise effects may exist in cancer with respect to activity of more recently evolved genes, in line with the recently proposed modification to the atavism hypothesis of a sequential loss of multicellular features in cancer. However, it was not our intent to capture such effects, which we consider out of the scope of this study.

It is worth noting recently evolved genes can have oncogenic roles as well. E.g., a new paper by Martins et al ([10.1158/0008-5472.CAN-23-1237](https://doi.org/10.1158/0008-5472.CAN-23-1237)) and the 2022 paper by Ma et al from *Genome Biology* we cite in our manuscript.

Thus, we focused our efforts on understanding how co-expression of unicellular and multicellular genes changes in cancer, rather looking for suppression of particular gene age categories, to gain insight into how loss of regulatory mechanisms supporting multicellularity contribute to cancer formation and progression.

Further, we have concerns it could be technically challenging to detect ‘mammalian-enriched’ co-expression modules because of the often low and irregular expression of mammalian genes in tumours we encountered in our previous work (PNAS 2017; eLife 2019).

2) The supplementary Data can not be found with the submission, and git hub repository link does not work.

We apologize for these omissions.

The Supplementary files have been uploaded with the revised manuscript.

We have verified the link provided in the manuscript (<https://github.com/cancer-evolution/Evolutionary_analysis_of_coexpression_modules>) is working. This repository contains the R object with gene assignments to modules (genes_assigned_to_modules.Rdata) needed to replicate the analyses and figures shown in our manuscript.

**Second round of review**

**Reviewer 1**

The authors have fully addressed my concerns. In particular, the added analysis on sc-RNA sequencing data strengthens their arguments.

**Reviewer 2**

All my concerns have been properly addressed, and I have no further comments.
